# Past, Present, and Future of Groundwater Remediation Research: A Scientometric Analysis

**DOI:** 10.3390/ijerph16203975

**Published:** 2019-10-18

**Authors:** Qibin Chen, Guilian Fan, Wei Na, Jiming Liu, Jianguo Cui, Hongyan Li

**Affiliations:** 1 College of Environmental Science and Engineering, Taiyuan University of Technology, Jinzhong 030600, China; 2 Faculty of Business Administration, Shanxi University of Finance and Economics, Taiyuan 030006, China; 3 Shanxi Province C&M Operation Center for Xishan Yellow River-lifting Irrigation Project, Taiyuan 030002, China

**Keywords:** groundwater remediation, research fronts, timeline, scientometrics, CiteSpace, visualization research

## Abstract

In this study, we characterize the body of knowledge of groundwater remediation from 1950 to 2018 by employing scientometric techniques and CiteSpace software, based on the Science Citation Index Expanded (SCI-E) databases. The results indicate that the United States and China contributed 56.4% of the total publications and were the major powers in groundwater remediation research. In addition, the United States, Canada, and China have considerable capabilities and expertise in groundwater remediation research. Groundwater remediation research is a multidisciplinary field, covering water resources, environmental sciences and ecology, environmental sciences, and engineering, among other fields. Journals such as *Environmental Science and Technology, Journal of Contaminant Hydrology*, and *Water Research* were the major sources of cited works. The research fronts of groundwater remediation were transitioning from the pump-and-treat method to permeable reactive barriers and nanoscale zero‑valent iron particles. The combination of new persulfate ion‑activation technology and nanotechnology is receiving much attention. Based on the visualized networks, the intelligence base was verified using a variety of metrics. Through landscape portrayal and developmental trajectory identification of groundwater remediation research, this study provides insight into the characteristics of, and global trends in, groundwater remediation, which will facilitate the identification of future research directions.

## 1. Introduction

Groundwater is an important natural resource that supports socioeconomic development and maintains ecological balance in modern societies [1]. It provides 36% of drinking water, 42% of water for agriculture, and 24% of water for industry [2,3]. The quality of groundwater resources globally is threatened by the natural geochemical background and anthropogenic pollution [4,5]. To clean polluted groundwater and ensure the sustainability of groundwater resources, a variety of remediation technologies (e.g., pump-and-treat, biodegradation, chemical oxidation, and reduction to adsorption) have been developed and applied [6,7,8,9]. Each treatment option has associated merits and demerits, depending on remedial goals and site conditions. There are several challenges for selecting sustainable remediation technologies and designing remediation strategies today, including evolving groundwater treatment goals, complex geophysical–chemical characterization, current understanding of available technologies, contaminant mixtures, and economic considerations [8,10,11,12].

Groundwater remediation research has been reviewed from a variety of perspectives, and the extant review articles focus on technologies for the remediation of contaminated groundwater and their applications. Examples include natural attenuation processes [13], permeable reactive barriers [14], sustainability appraisal tools [15], nanoscale zero‑valent iron particles [16], and iron sulphide particles for groundwater remediation [17]. Nevertheless, little attention has been devoted to quantitative analyses of the evolution of groundwater remediation research. In short, they did not provide an overall landscape of the groundwater remediation literature. In a recent article, Zhang, Mao, Crittenden, Liu and Du [8] used social network analysis and bibliometric technology to evaluate publications related to groundwater remediation from 1995 to 2015, and the results provided valuable insight into groundwater remediation. However, they did not identify and evaluate hotspots and there is, to date, no knowledge base for groundwater remediation.

This study has used scientometric approaches to describe the development trajectory and landscape of groundwater remediation research quantitatively and systematically, and the research frontiers and emerging trends of the groundwater remediation literature were detected and identified using the visualization tool CiteSpace. The results will provide a useful reference for academics, researchers, and policy decision makers.

## 2. Data Acquisition and Methods

### 2.1. Data Acquisition

It is essential for researchers to quickly and accurately locate publications, using the search strategy and screen methods. The article retrieval source for analysis was the SCI-E databases, which are frequently used in scientific research [18]. Several topic terms, including “groundwater restoration”, “groundwater reclamation”, and “groundwater remediation”, were used to retrieve publications that contained these words in publications’ titles, keyword lists, and abstracts. These terms helped to locate the majority of groundwater-remediation-related publications. Though there may be other groundwater-remediation-related terminology, they account for a small percentage of publications and may have marginal relation to groundwater remediation research [19]. The search results have been refined or filtered by web of science categories, research areas, and document types. To do this, several categories needed to be excluded, such as (i) unrelated categories—physiology, pharmacology pharmacy, genetics heredity—(ii) document types—book chapter, data paper, proceedings abstract—and (iii) research areas—imaging science photographic technology, business economics. The search resulted in 2867 publications dated from January 1950 to September 2018. The entire records, including the titles, abstracts, keywords, and references, were then exported for subsequent analyses. Based on the frequency of groundwater remediation research over the past seven decades, we reviewed numerous studies published between 1950 and 2018 (see the Supplementary Materials).

### 2.2. Scientometric Analytical Methods

Scientometrics, created by Tibor Braun [20], has been defined as the “quantitative study of science and technology” [21]. As a branch of informatics, scientometrics is used to analyze patterns in scientific literature quantitatively, to understand the knowledge structure of emerging trends in a research field [22]. As a scientometric approach, CiteSpace is used to clarify multidisciplinary relationships, assess research status, map knowledge domains, reveal research frontiers, and predict emerging trends, by analyzing the bibliographic records of associated publications [23]. In the net knowledge maps generated by CiteSpace, a node represents one item (e.g., country, author, subject, term, journal, or reference), and links describe co-citations or co-occurrences between these nodes [24]. Furthermore, each node is described as a series of tree rings of different colours, with blue representing the oldest and orange the most recent [25]. A purple ring around an item indicates good centrality.

To date, CiteSpace has been employed in studies of, for example, nonpoint source pollution [26], information sciences [27], psychological science [28], and global green innovation [29]. In this study, we produced and analyzed co-occurrence networks of subject categories and countries, and co-citation networks of journals, authors, and references using CiteSpace.

## 3. Results and Discussion

### 3.1. Characteristics of Publication Output

**Figure 1:**
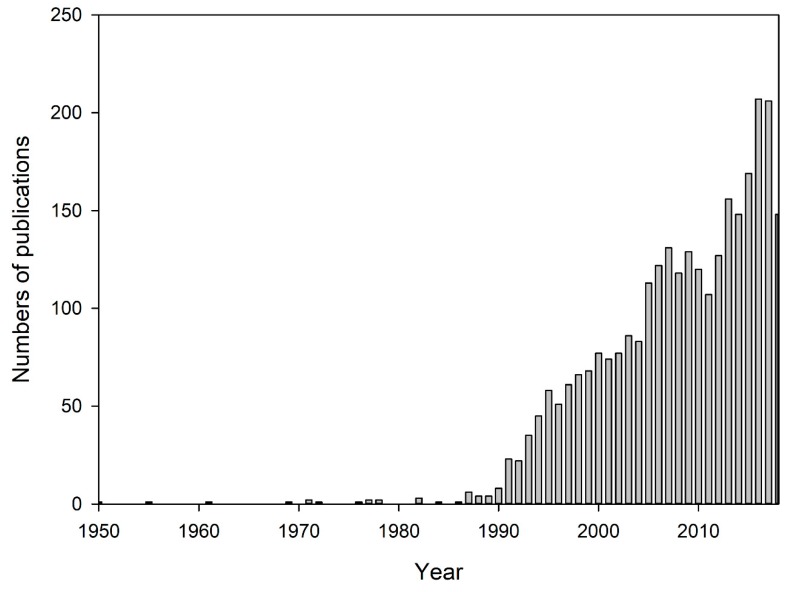
Publication output performance during the period 1950–2018.

To give an overview of research in groundwater remediation, the annual number of articles published from 1950 to 2018 (total, 2867) is shown in Figure 1. In 1950, only one article, titled “Ground-Water Pollution in Michigan”, was published [30]. Subsequently, the annual number of publications was fewer than 10 until 1990. The number of articles increased significantly after this period, from 23 in 1991 to 207 in 2016.

### 3.2. Co-Operations of Countries/Territories and Institutions

#### 3.2.1. Co-Operation among Countries/Territories

**Figure 2:**
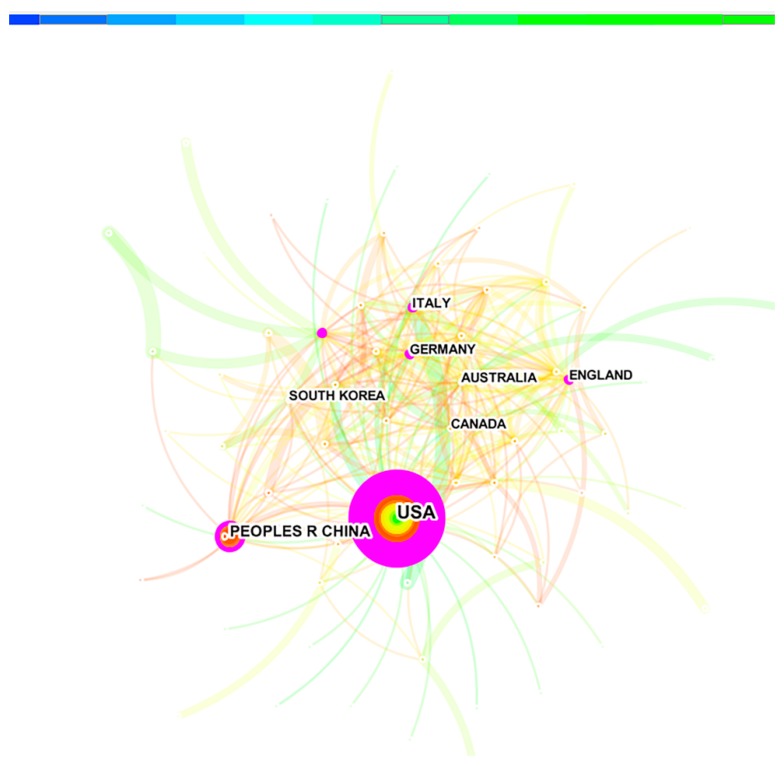
Distribution of co-operation among countries/territories.

Running CiteSpace, we obtained a countries/territories distribution with 78 nodes and 296 links (Figure 2); this map can help researchers find their colleagues elsewhere in the world and establish collaborations. Each node represents a different country or territory, and the size of the node represents the number of publications. Similarly, the lines connecting countries/territories indicate their co-operation, while the thickness of the line represents the degree of co-operation [8]. The United States (U.S.) was the hub of the co-operation network, and the leader in groundwater remediation research, in collaboration with other productive countries/territories. The discovery of hazardous waste at Love Canal in Niagara, New York, and many other places in the United States, heralded a new era in hazardous waste problems by the end of the 1970s. Subsequently, civil and environmental engineers, hydrologists, hydrogeologists, and other scientists became involved in the identification, evaluation, and remediation of groundwater-contaminated hazardous waste sites [31]. Groundwater remediation research was distributed among 78 countries/territories, and the top 10 countries/territories published 2607 articles, accounting for 90.9% of the total (Table 1). The U.S. and China published 1152 and 464 articles, respectively, ranking first and second, and accounting for 56.4% of total articles. Thus, the U.S. and China were two major research powers in groundwater remediation and far ahead of other countries/territories.

#### 3.2.2. Co-Operation among Institutions

**Figure 3:**
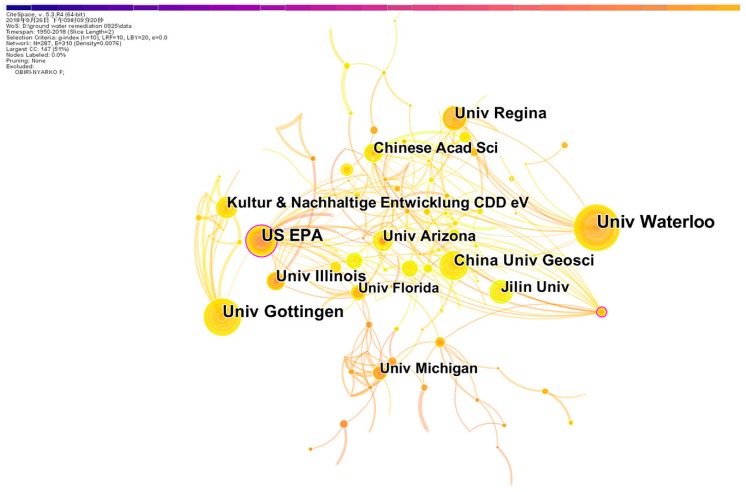
Distribution of co-operation among research institutions.

Institutional co-operation was also analyzed using CiteSpace (Figure 3). The top 10 productive institutions are shown in Table 1. These 10 productive institutions worked closely with organizations in geographical proximity, e.g., the University of Waterloo and University of Regina in Canada, the University of Illinois and the University of Arizona in the U.S., and China University of Geosciences and Jilin University in China. Therefore, it is necessary to strengthen international co-operation in the future.

The top 10 research institutions issued 455 articles, accounting for 15.9% of the total. According to Table 1 and Figure 3, the first major research echelon was led by the University of Waterloo, where hydrologists first used zero-valent iron (Fe^0^) to treat contaminated groundwater in situ approximately three decades ago [32,33]. Of these top 10 institutions, three were in the U.S. and three in China, confirming that the U.S., Canada, and China have considerable capabilities in groundwater remediation research, and strong expertise in research and development.

### 3.3. Co-Occurrence of Subject Categories

**Figure 4:**
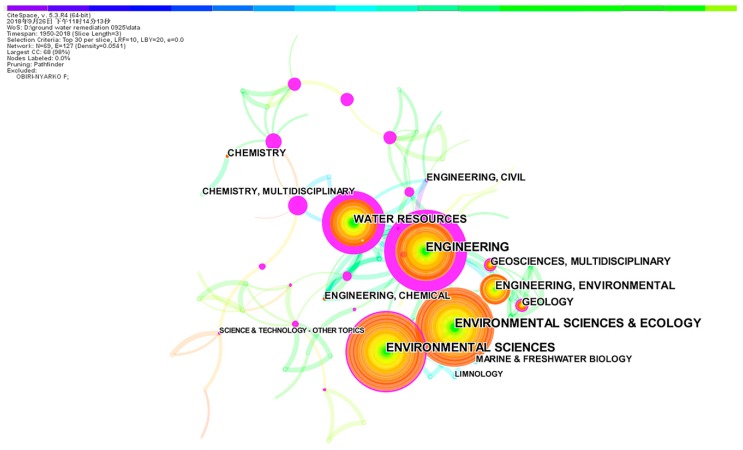
Disciplines involved in the field of groundwater remediation, shown as a network of subject categories.

Based on co-occurrence analyses of subject category, the disciplines involved in groundwater remediation can be detected. In this study, we selected the top 30 subject categories with the largest number of reoccurrences each year for category characteristic analysis. The information on subject categories was extracted from the SCI-E databases using CiteSpace and analyzed. Figure 4 shows the co-occurrence network from 1950 to 2018, where one node represents a subject category, and the edge connecting two nodes represents the co-occurrence of two subject categories. The top three popular research categories were environmental sciences and ecology, environmental sciences, and engineering. Of the top 10 subject categories, engineering had the central position and played an important role in groundwater remediation. Material science was second, followed by water resources, and chemistry, physical. Therefore, groundwater remediation is a multidisciplinary research field, involving an extensive range of interests.

### 3.4. Journal Citation Analyses

**Figure 5:**
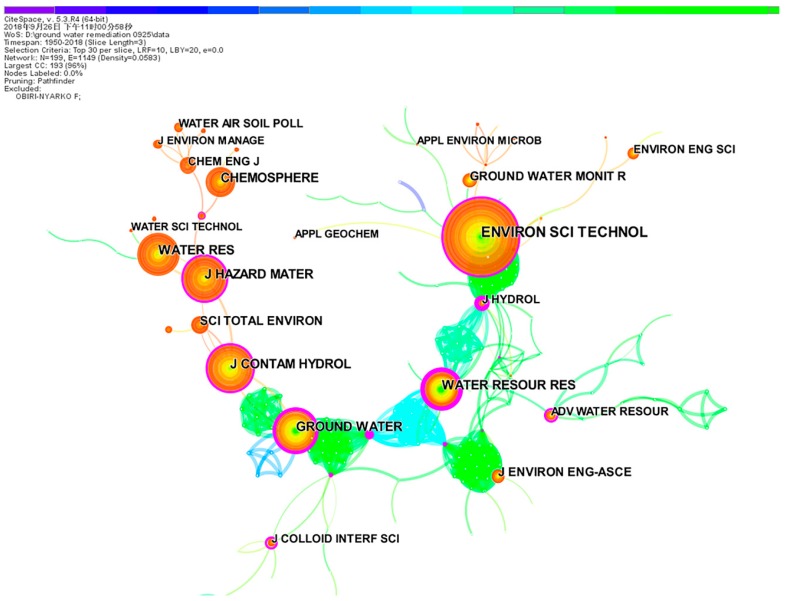
Journal co-citation knowledge map.

“Core journals” usually refer to top-ranking journals with high citation frequencies. We produced a groundwater remediation journal co-citation map with 199 nodes and 1149 links, using CiteSpace software (Figure 5). The top 10 most productive journals, with >500 citations, are listed in Table 2. The total of 12,090 citations from the 10 journals accounts for 34.74% of the total citation count. Thus, the citation distribution was concentrated. In addition, these 10 journals were defined as the “core journals” in the field of groundwater remediation.

*Environmental Science and Technology* and *Water Research* were the core journals in groundwater remediation research, with 1850 and 1204 citations, respectively (Table 2). *Water Research* and *Environmental Science and Technology* also had the highest IFs, at 7.051 and 6.653, respectively.

### 3.5. Author Citation Analyses

**Figure 6:**
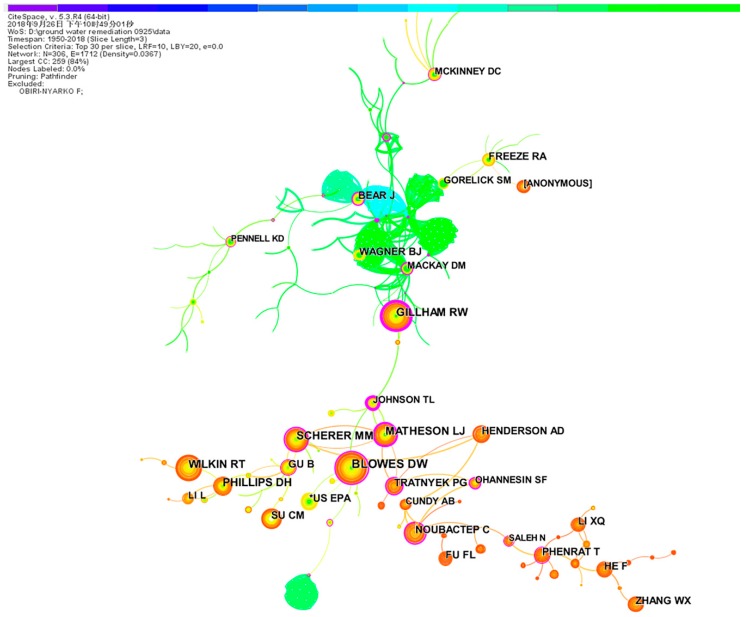
Author co-citation map/the co-operation network of productive authors.

White and McCain [34] first proposed the author co-citation concept in the U.S. Author co-citation maps, which reflect the closeness of the research directions and importance of the authors, and have been widely used to assess scientific research ability and relevance. Herein, one node represents a cited author, and an author co-citation knowledge map was created using CiteSpace (Figure 6).

The largest node corresponded to Blowes DW, whose articles were cited 251 times; this was followed by Gillham RW (233), Wilkin RT (204), Matheson LJ (190), Scherer MM (177), Phillips DH (165), Su CM (157), and Noubactep C (145). Thus, these authors’ works had a marked impact on groundwater remediation research and development, and they represent the core research strength in the field.

As mentioned above, the first major research echelon, led by the University of Waterloo, was composed of Gillham RW, Blowes DW, and other authors, suggesting that this group had the greatest impact on groundwater remediation research.

### 3.6. Documents Co-Citation Analyses

**Figure 7:**
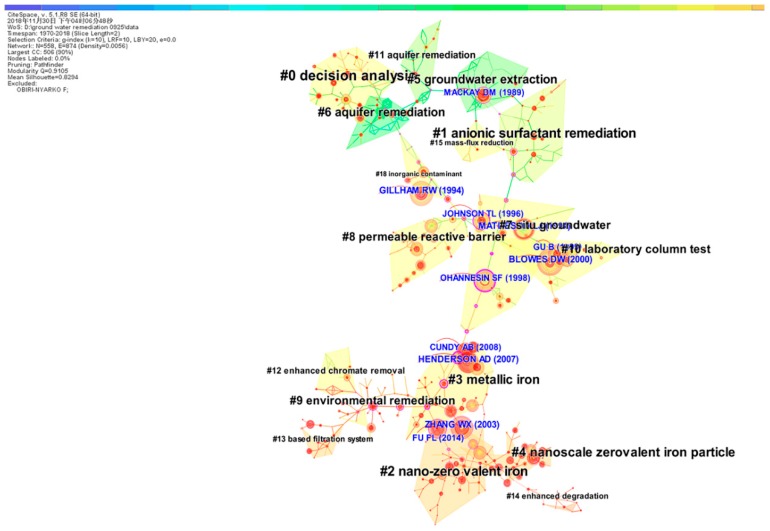
The synthetic network of co-cited references.

The co-citation network was divided into many clusters of co-cited references in CiteSpace, so that references are closely connected within the same cluster but loosely connected among different clusters (Figure 7). The 10 major clusters are listed in Table 3 by size, which represents the number of members in each cluster. The silhouette score of a cluster reflects its quality, i.e., homogeneity or consistency. If the silhouette value of a cluster is close to 1.0, then it was homogenous [22]. All the clusters in Table 3 were highly homogeneous, as indicated by their high silhouette scores. Noun phrases from the terms (e.g., titles or abstracts) used in articles in the cluster were used to label each cluster. Labels selected by the log-likelihood ratio (LLR) test were used in subsequent discussions [35].

We can identify the average year of the publications in a cluster by their recentness, i.e., Cluster #6 on aquifer remediation had an average year of publication of 1985. The recently formed clusters, Clusters #2 and #3 (nano-zero‑valent iron and metallic iron, respectively), had an average year of publication of 2009 and 2008, respectively.

#### 3.6.1. Analyses of Research Fronts

Price [36] proposed the concept of the research front, and postulated that a research front can characterize the momentary nature of a research field. Garfield [37] defined a research front as “a cluster of co-cited articles and all articles that cite the cluster”. Chen [38] defined a research front as “an emergent and transient grouping of concepts and underlying research issues”. CiteSpace shapes the network knowledge map of research fronts, with mutant terms that can be extracted from the index terms, abstracts, titles, and record indicators of the references. Specific methods include selecting a cited reference as the net node, the g-index (*k* = 10) as threshold willing, and the key pathfinder algorithm. We obtained 17 clusters by selecting “Find clusters” and abstracted the names of the clusters by selecting “Label clusters with indexing terms”. Figure 7 shows the net knowledge map generated.

There were 558 nodes, 874 links, and 17 clusters. Clusters #2, #3, and #4 had a high concentration of nodes with citation bursts, which echoed the fact that these were the most recently formed clusters.

If a cluster has a larger area, it has more bibliographic entries, and large clusters generally indicate main research directions, i.e., each cluster corresponds to a research front. The research fronts and major trends in groundwater extraction, in situ groundwater remediation, permeable reactive barriers, metallic iron, and nanoscale zero‑valent iron particles are shown in Figure 7 and Table 3.

#### 3.6.2. Timeline of Research Fronts

**Figure 8:**
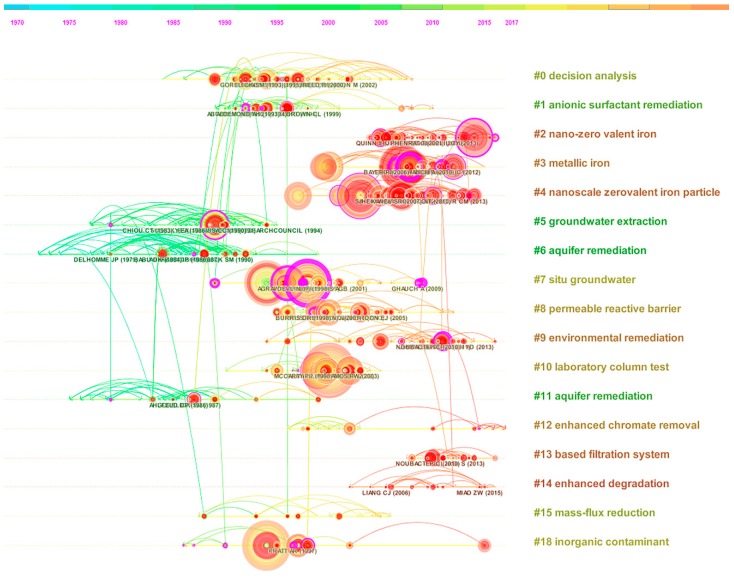
Timelines of co-citation clusters. Major clusters are labelled on the right.

Figure 8 shows timelines of the 17 distinct co-citation clusters and their interrelationships. All timelines run from left to right [39], show the times at which research fronts appear and disappear, and display structural information about the research front clusters [40]. Analysts can visually identify various characteristics of a cluster, such as its citation classics, historical length, citation bursts, and connection to other clusters.

The following paragraphs provide an interpretation of the research fronts’ timelines. Groundwater remediation research has a long history (Figure 8). The earliest research front, “aquifer remediation”, provided basic information for subsequent research. Next, technologies to deal with contaminated groundwater were developed. The containment and/or control of contaminated groundwater can generally be accomplished using one, or a combination, of several available techniques, which can be broken down into aquifer rehabilitation, physical containment measures, and withdrawal, treatment, and use [41].

The second research front, “groundwater extraction”, began around 1979, and pump‑and‑treat as a groundwater extraction technology began at selected sites in 1982 [42], in response to groundwater pollution control and contamination remediation. Earlier pump‑and‑treat systems, which did not consider the presence of geologic heterogeneity, poor definition of initial condition in source zones, did not clean aquifers to the required level. Many of the original systems worked adequately for a period of time, but, after they were switched off, the contaminant levels at many sites reached values higher than those before remediation [31]. Subsequent to the pivotal 1989 article by Mackay [43], the research front became inactive. A number of new technologies for groundwater remediation are under development, and these may accelerate contaminant removal from the subsurface (e.g., injection of steam, surfactants) or destroy the contaminant in situ [43]. Hence, research fronts are discontinuous, and start and end abruptly when scientists move from one puzzle to the next [40].

In the 1990s, scientists and engineers had to prepare to deal with recent puzzles, which included residual oils, source zones with non-aqueous phase liquids (NAPLs), and vapours in the unsaturated zone [31]. During this period, four research fronts, “anionic surfactant remediation (1995)”, “decision analyses (1997)”, “laboratory column test (1999)”, and “in situ groundwater remediation (1999)”, were created in response to growing concern over efficient and cost-effective clean‑up solutions. Among these research fronts, “in situ groundwater remediation” was worthy of note. This research front experienced a period of stability and extends to the present. A growing number of researchers focused on the development of in situ remediation technologies, e.g., in situ chemical oxidation [ISCO]. ISCO, a type of advanced oxidation process technology, has proven useful for in situ remediation technology for the most prevalent organic contaminants in groundwater. The development of in situ remediation technologies led to the formation of three research fronts in the new century: “permeable reaction barriers (PRBs) (2002)”, “metallic iron (2008)”, and “nano zero‑valent iron (nZVI) (2009)”.

The research front, “PRB”, which dates to 1989, had a median publication date of 2002. Over the last two decades, PRBs have been emerging as an effective alternative passive in situ remediation technology. In the 1990s, research on PRBs increased considerably, which led to many new approaches for suitable reactive materials, target contaminants, and PRB design.

“nZVI” is the latest research frontier, showing rapid growth and a professional pattern. Gillham and O’Hannesin [44] discovered that halogenated aliphatic compounds in groundwater can be reduced using bulk ZVI. This characteristic of iron led to the advanced Fe-PRB, in which vertical trenches were filled with granular ZVI, placed in the flow path of the underground contaminant plumes [45,46].

A report [47] by the Chinese Academy of Sciences indicated that the third top research front in ecology and environmental sciences, entitled “Activation of persulfate for degradation of aqueous pollutants by transition metal and nanotechnology”, is receiving much global attention. The combination of persulfate ion activation technology and nanotechnology will improve the efficiency of polluted water treatment, reduce energy consumption, and promote recycling.

#### 3.6.3. Analyses of the Intelligence Base

Chen [38] defined “the intellectual base of a research front as its citation and co-citation footprint in the scientific literature, an evolving network of scientific publications cited by research-front concepts”.

(1) Most‑Cited Articles

The most‑cited articles are generally considered landmarks, owing to their ground‑breaking contributions [22]. Cluster #7 had three of the top 10 landmark articles, and Clusters #3 and #10 each had two (Table 4). The most-cited articles in the databases were by Blowes (2000), with 154 citations, followed by Gillham (1994), with 144 citations and Matheson (1994), with 135 citations, and the most recent was a review article by Fu (2014). Interestingly, the titles of the most‑cited articles contained the terms “permeable reactive barriers”, “zero-valent iron”, “nanoscale iron particles” (Table 4), which were in accordance with the research fronts noted above.

Gillham and O’Hannesin [44] investigated the potential of Fe^0^ in the dehalogenation of ethanes, ethenes, and 14 chlorinated methanes. The results demonstrated biotic reductive dechlorination, in which iron serves as the source of electrons. In response to the rapid degradation rates, an application for in situ remediation of contaminated groundwater was proposed.

Blowes, et al. [48] was cited the most frequently. This paper reviewed the recent research progress in PRBs for the remediation of inorganic contamination of groundwater.

(2) Betweenness Centrality

The betweenness centrality measure that Freeman [49] proposed is used to give prominence to potential pivotal points in the synthesized network shifts over time. The betweenness centrality of nodes in a network is indicative of the importance of the location of the nodes. We are especially interested in the nodes located between different node groups, because they probably offer insight into emerging trends [22]. Table 5 shows 10 structurally crucial references in the network, and three of these nodes were in Cluster #3, and five in Cluster #7. These references can be identified as landmark works in the field of groundwater remediation.

(3) Citation Bursts

**Figure 9:**
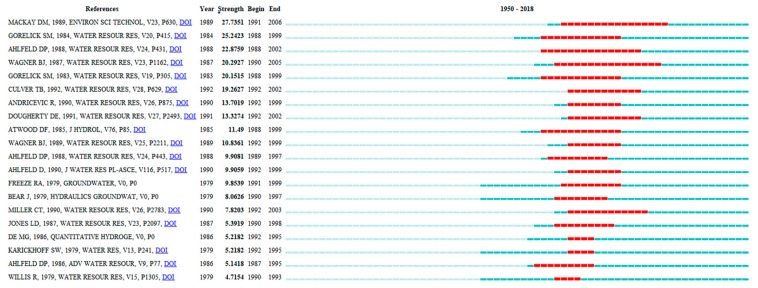
The top 20 references with the strongest citation bursts.

A reference citation burst may indicate an emergent research front, and the citation-burst-detection algorithm of Kleinberg [50] is adapted for identifying emergent research front concepts. Table 6 lists the references that had the strongest metric of citation bursts across the entire database during the period 1950–2018. Among the articles with strong citation bursts (Table 6 and Figure 9), Mackay and Cherry [43] is worthy of note. Their article explored the reasons for the difficulty of groundwater clean-up, noted some implications, and suggested that achieving stringent health-based clean-up standards is unlikely, and the ultimate cost of clean-up is high in many cases. Thus, they suggested that site characterization and remediation have much room for improvement, by both the development of new tools and ongoing training of staff [43]. Subsequently, the development of permeable reactive barrier technology using zero-valence iron filings has proceeded from recognition, evaluation, technology conceptualization, and proof of concept, to commercialization.

(4) Sigma

The structural centrality and citation burstness of cited references can be measured by the Sigma metric measure, i.e., the Sigma value of a reference that is strong in both measures will be higher than that of a reference that is strong in only one of the two measures [22] (Table 7). The pioneering article by Fu, et al. [51] had the highest Sigma of 101,578.09, indicating it to be structurally indispensable in the field, due to its strong citation burst. This article reviewed the recent advances of ZVI and the progress made in groundwater remediation using ZVI technology.

## 4. Conclusions

### 4.1. Summary

This study offers a comprehensive scientometric review of groundwater remediation research. There were 2867 journal articles related to this field published from 1950 to 2018, and the increasing annual number of publications suggests a continued research interest and a globally urgent need to remediate contaminated groundwater, since 1991. The U.S. and China contributed 56.4% of the publications and were the major powers in groundwater remediation research. Groundwater remediation research is a multidisciplinary research field and covers an extensive range of interests, from environmental sciences and ecology to environmental sciences, engineering, and water resources. Furthermore, journals such as *Environmental Science and Technology*, *Water Research*, and *Journal of Contaminant Hydrology* were the main sources of cited works in groundwater remediation research. The research fronts of groundwater remediation were transitioning from the pump-and-treat method to PRBs and nanoscale zero‑valent iron particles. The combination of persulfate ion activation technology and nanotechnology shows promise. Meanwhile, based on the visualized networks, the intelligence base was verified using a variety of metrics. Our study provides a valuable reference for researchers in the field of groundwater remediation, and others with interests in this area.

### 4.2. Future Outlook

(1) Development of treatment trains. Great advances have been made in the field of groundwater remediation research over recent decades. As the “One Size Fits All” remedy technology does not work effectively at most contaminated sites, the groundwater remediation technologies used are generally parts of a “treatment train”. Hence, tailored approaches and remediation techniques on a site-by-site basis are needed. In addition, research into technologies for pollution remediation of fractured bedrock aquifers, low permeability formations, and green remediation technology, is needed. Hence, these topics will remain areas of active research for many years.

(2) Optimization of groundwater remediation design under uncertainty. The technical and environmental challenges in designing optimal groundwater remediation systems are the spatial variability of natural aquifers, uncertain aquifer parameters, and complex site characteristics, which affect both the cost and efficiency of remediation. Investment in data collection and accurate site characterization may minimize uncertainty. Simultaneously, effort has been made to include uncertainty analyses in optimal groundwater remediation designs, using optimization methods and a coupled simulation–optimization approach. Owing to the complexity and inherent uncertainty of groundwater remediation technologies, the success of their field application is limited. This suggests the need to incorporate new methods and means of quantitatively analyzing uncertainty into the design of optimal groundwater remediation technologies.

(3) Development of green and sustainable remediation. Green and sustainable remediation (GSR) is a new movement in the land and groundwater remediation field that has drawn much attention globally in recent years, and it requires consideration of the environmental, economic, and social dimensions of sustainability. GSR technologies for contaminated groundwater, including biochar materials, green synthesis of engineered nanoparticles [52], sustainable PRB [53] and sustainably released long-term green remediation materials, have made rapid progress. However, case studies indicate that public participation must be improved to promote social sustainability, and region-specific factors should be considered when implementing GSR.

## Figures and Tables

**Table 1:** Distribution of 10 co-operative countries/territories and institutions.

Ranking	Publications	Country/Territory	Publications	Institution
1	1152	USA	72	University of Waterloo
2	464	People’s Republic of China	57	University of Gottingen
3	212	Canada	57	U.S. EPA
4	200	Germany	46	China University of Geosciences
5	115	Italy	40	University of Illinois
6	111	South Korea	40	University of Regina
7	98	Australia	37	University of Arizona
8	94	England	37	Chinese Academy of Sciences
9	83	Taiwan	35	Kultur und Nachhaltige Entwicklung
10	78	India	34	Jilin University

**Table 2:** Distribution of 10 “core journals” and IF in 2017.

Ranking	Citation Count	Journal Title	Publication Year	IF (2017)
1	1850	Environmental Science & Technology	1967	6.653
2	1204	Water Research	1967	7.051
3	1203	Journal of Contaminant Hydrology	1986	2.284
4	1169	Journal of Hazardous Materials	1975	6.434
5	1107	Ground Water	1963	1.900
6	1017	Water Resources Research	1965	4.361
7	933	Chemosphere	1972	4.427
8	556	Journal of Environmental Engineering-ASCE	1983	1.396
9	553	Science of The Total Environment	1972	4.610
10	537	Ground Water Monitoring and Remediation	1981	1.648

**Table 3:** Major clusters of co-cited references.

Cluster ID	Size	Silhouette	Label (MI)	Label (LLR)	Year Ave.
0	46	0.909	optimization	decision analysis	1997
1	42	0.940	remediation	anionic surfactant remediation	1995
2	42	0.927	zero-valent iron	nano-zero-valent iron	2009
3	39	0.957	zero-valent iron	metallic iron	2008
4	38	0.909	groundwater remediation	nanoscale zero valent iron particle	2008
5	37	0.949	remediation	groundwater extraction	1988
6	36	0.960	remediation	aquifer remediation	1985
7	33	0.912	zero-valent iron	in situ groundwater remediation	1999
8	33	0.966	permeable reactive barrier	permeable reactive barrier	2002
10	31	0.920	permeable reactive barrier	laboratory column test	1999

MI = mutual information, LLR = log-likelihood ratio, Ave = average.

**Table 4:** The top 10 most-cited references.

Citation Count	Title	First Author	Year	Source	Cluster #
154	Treatment of Inorganic Contaminants using Permeable Reactive Barriers	Davie W. Blowes	2000	Journal of Contaminant Hydrology	10
144	Enhanced Degradation of Halogenated Aliphatics by Zero-Valent Iron	Robert W. Gillham	1994	Ground Water	18
135	Reductive Dehalogenation of Chlorinated Methanes by Iron Metal	Leah J. Matheson	1994	Environmental Science and Technology	7
130	Nanoscale Iron Particles for Environmental Remediation: An Overview	Wei-Xian Zhang	2003	Journal of Nanoparticle Research	4
130	Long-Term Performance of Zero-Valent Iron Permeable Reactive Barriers: A Critical Review	Andrew D. Henderson	2007	Environmental Engineer Science	3
122	Long-Term Performance of an in situ “Iron Wall” for Remediation of VOCs	Stephanie F. O’Hannesin	1998	Ground Water	7
105	Biogeochemical Dynamics in Zero-Valent Iron Columns Implications for Permeable Reactive Barriers	B. Gu	1999	Environmental Science and Technology	10
99	Colloid Transport in Geochemically Heterogeneous Porous Media Modeling and Measurements	Philip R. Johnson	1996	Environmental Science and Technology	7
99	The Use of Zero-Valent Iron for Groundwater Remediation and Wastewater Treatment: A Review	Fenglian Fu	2014	Journal of Hazardous Materials	2
97	Use of Iron-Based Technologies in Contaminated Land and Groundwater Remediation: A Review	Andrew B. Cundy	2008	Science of the Total Environment	3

**Table 5:** Cited citations with the highest between centrality.

Rank	Centrality	Reference	Cluster #
1	0.95	Devlin JF, 1998, Environ Sci Technol, V32, P1941	7
2	0.82	Ohannessin SF, 1998, Groundwater, V36, P164	7
3	0.81	Noubactep C, 2008, Environ Technol, V29, P909	3
4	0.80	Noubactep C, 2009, J Hazard Mater, V166, P79	7
5	0.80	Balko BA, 1998, J Phys Chem B, V102, P1459	7
6	0.79	Kang SH, 2009, Environ Sci Technol, V43, P3966	7
7	0.78	Noubactep C, 2007, Open Environmental Sciences, V1, P9	3
8	0.69	Noubactep C, 2011, Water Sa, V37, P419	3
9	0.52	Su CM, 1999, Environ Sci Technol, V33, P163	8
10	0.47	West CC, 1992, Environ Sci Technol, V26, P2324	1

**Table 6:** The top five references with the strongest metric of citation bursts.

Rank	Citation Bursts	Reference	Duration	Cluster #
1	27.74	Mackay DM, 1989, Environ Sci Technol, V23, P630	1991–2006	5
2	25.24	Gorelick SM, 1984, Water Resour Res, V20, P415	1988–1999	6
3	22.88	Ahlfeld DP, 1988, Water Resour Res, V24, P431	1988–2002	6
4	20.23	Wagner BJ, 1987, Water Resour Res, V23, P1162	1990–2005	11
5	20.15	Gorelick SM, 1983, Water Resour Res, V19, P305	1988–1999	11

**Table 7:** Structurally and temporally significant references.

Sigma	Burst	Centrality	Citations	Reference	Cluster #
101,578.09	38.0	0.35	99	Fu FL, 2014, J Hazard Mater, V267, P194	2
13,386.93	16.54	0.78	41	Noubactep C, 2007, Open Environmental Sciences, V1, P9	3
2829.78	13.45	0.81	61	Noubactep C, 2008, Environ Technol, V29, P 909	3
1380.75	27.60	0.30	95	Mackay DM, 1989, Environ Sci Technol, V23, P630	5
290.08	10.85	0.69	24	Noubactep C, 2011, Water Sa, V37, P419	3

## Supplementary Materials

Publications list for scientometric analysis (topic searched results in SCI-E database) includes title of the article, journal name, authors, year of publication, volume, page range. The following is available online at https://www.mdpi.com/1660-4601/16/20/3975/s1, Spreadsheet: Publications list.
